# Volume Changes in Brain Subfields of Patients with Alzheimer’s Disease After Transcranial Ultrasound Stimulation

**DOI:** 10.3390/diagnostics15030359

**Published:** 2025-02-04

**Authors:** Sheng-Yao Huang, Meng-Ting Wu, Chung-Fu Sun, Feng-Yi Yang

**Affiliations:** 1 Department of Mathematics, Soochow University, Taipei 111, Taiwan; sy_huang@scu.edu.tw; 2 Division of Neurosurgery, Cheng Hsin General Hospital, Taipei 111, Taiwan; m860747@gmail.com; 3 Department of Biomedical Imaging and Radiological Sciences, National Yang Ming Chiao Tung University, Taipei 111, Taiwan; iamtonyinnthu@gmail.com

**Keywords:** transcranial ultrasound stimulation, Alzheimer’s disease, cognitive function, MRI, brain volume

## Abstract

**Background/Objectives**: Alzheimer’s disease (AD) is characterized by progressive brain atrophy marked by cognitive decline and memory loss, which significantly affect patients’ quality of life. Transcranial ultrasound stimulation (TUS) is a potential physical treatment for AD patients. However, the specific brain regions stimulated by TUS and its therapeutic effects remain unclear. **Methods**: In this study, magnetic resonance imaging (MRI) and FreeSurfer segmentation were employed to assess alterations in the brain volume of AD patients after TUS. **Results**: Our findings revealed significant volume increases in the corpus callosum (CC) and lateral orbitofrontal cortex (lOFC) in the TUS group. Moreover, the volumetric changes in the CC were strongly correlated with improvements in the Mini-Mental State Examination score, which is a widely used measure of cognitive function of AD patients. **Conclusions**: TUS has the potential to alleviate disease progression and offers a non-invasive therapeutic approach to the improvement of cognitive function in AD patients.

## 1. Introduction

The primary approach to treating Alzheimer’s disease (AD) currently involves pharmacotherapy. Cholinesterase inhibitors, such as donepezil (Aricept^®^), rivastigmine (Exelon^®^), and galantamine (Reminyl^®^), are commonly used to treat mild AD [1], whereas N-methyl-D-aspartate (NMDA) receptor antagonists, such as memantine (Ebixa^®^, Witgen^®^, and Manotin^®^), are employed for moderate-to-severe cases [2]. However, the efficacy of pharmaceutical interventions is limited, and their use may be associated with side effects, such as nausea, vomiting, dizziness, diarrhea, headache, and constipation [3]. This study explored a novel alternative therapy using low-intensity pulsed ultrasound (LIPUS) to physiologically stimulate the brain and induce the production of endogenous brain-derived neurotrophic factor (BDNF) [4,5]. The aim was to use LIPUS to achieve therapeutic effects for AD patients while reducing the side effects associated with drug therapy.

In vitro and in vivo experiments have demonstrated that ultrasound stimulation promotes the expression of endogenous neurotrophic factors, including BDNF, glial cell-line-derived neurotrophic factor, and vascular endothelial growth factor, in astrocytes [4,6]. Preclinical animal studies targeting AD and vascular dementia models suggest that transcranial ultrasound stimulation (TUS) increases the protein expression of neurotrophic factors, leading to improvements in cognitive behavior. This performance has been validated through pathological immunostaining and positron emission tomography imaging, with no apparent physiological harm or inflammatory reactions observed. This method aids in protecting brain tissue and repairing nerve cells [5,7,8].

The degree of brain volume atrophy in AD patients can vary in different brain regions. Magnetic resonance imaging (MRI) can reveal various patterns of brain volume changes in AD, including volume decreases, increases, or no changes in specific brain regions [9,10,11,12]. In contrast, studies using manual and semi-automated methods with control volunteer researchers found a decrease in hippocampal volume and an increase in the temporal horn volume in AD patients [13,14]. Brain volumes can be quantified using either manual or automated methods [15,16].

Compared to healthy brains, AD patients’ brains show atrophy in the hippocampus and entorhinal cortex regions in MRI scans [17]. This study focused on analyzing the changes in brain volume, as revealed by MRI, in AD patients after physiological stimulation with LIPUS. For volume segmentation, we employed the automated segmentation technique, FreeSurfer, commonly used in previous works, to delineate the differences in brain regions between the LIPUS treated AD group and a placebo group. We identified statistically significant reductions and increases in certain brain regions related to the disease in the AD group after LIPUS stimulation compared to the placebo group. Our results provide evidence that LIPUS stimulation can have an impact on brain volume in AD patients.

## 2. Materials and Methods

### 2.1. Study Design and Participants

The trial protocol was implemented in accordance with the principles of Good Clinical Practice guidelines and was approved by the Institutional Review Board of the Taipei Veterans General Hospital (IRB-TPEVGH No: 2018-06-007B#3) and Taiwan Food and Drug Administration (IRB No: 1086018000). This study is registered with ClinicalTrials.gov, number NCT03896698. We recruited patients aged 55–90 years with mild AD, determined based on NIA/AA criteria, with Mini-Mental State Examination (MMSE) scores ranging from 20 to 26. The inclusion criteria are shown in Table 1. The safety and efficacy of ultrasound brain stimulation were evaluated in terms of cognitive function using MRI and MMSE. The MMSE scores range from 0 to 30, with lower scores indicating greater cognitive impairment.

### 2.2. Sample Size

The estimated sample size was 20 subjects on a randomization ratio of 3:1 (treatment/control). The final analytical population included nine subjects, comprising six subjects in the treatment group and three subjects in the placebo group. The placebo group wore the same ultrasound device as the treatment group but received no stimulation.

### 2.3. Ultrasound Equipment

This study was a randomized, double-blind, placebo-controlled trial of LIPUS treatment for patients with mild AD. The experiment employed a non-invasive LIPUS device operating at 1.0 MHz, specifically designed for TUS with two plane probes. The device has passed electrical safety and electromagnetic compatibility tests. The participants wore an ultrasound stimulation helmet, and the ultrasound treatment probes, coated with ultrasound-conductive gel, made contact with the target area (near the left and right temples). The ultrasound probe on the helmet delivered ultrasound to the brain region (Figure S1). TUS was applied at a spatial-peak temporal-average intensity (ISPTA) of 500 mW/cm^2^, in adherence to the safety regulations set by the US Food and Drug Administration (ISPTA below 720 mW/cm^2^). The total duration of the TUS treatment was six weeks. Each treatment session included three episodes of ultrasound stimulation, each lasting for 5 min, for a total of 15 min per treatment session (per day). The subjects received 1 session per day, five days within each weekly cycle, for a total of 30 treatment sessions over the six-week treatment duration.

### 2.4. Safety Analyses Using MRI

Before and after TUS treatment, MRI scanning (3T MRI, Discovery™ MR750, GE healthcare, Waukesha, USA) was performed and included the following sequences: T1- and T2*-weighted imaging, fluid-attenuated inversion recovery (FLAIR), susceptibility-weighted imaging, and diffusion-weighted imaging. Ultrasound has been shown to transiently disrupt the blood–brain barrier (BBB) without obvious tissue damage. Therefore, BBB integrity was evaluated using T2-weighted gadolinium-enhanced MRI. The incidence of cerebral abnormality, indicated by pre-defined symptoms (i.e., cerebral atrophy, symptomatic intracerebral hemorrhage, and structural brain disorder) assessed by MRI and dynamic contrast-enhanced MRI, was obtained for both the treatment and the placebo group at each assessment time point. Abnormality was defined as any clinically significant abnormal finding. A shift in clinical significance, from baseline to the last assessment, was determined for each pre-defined cerebral abnormality symptom as the number and percentage of subjects with the shift in each group.

### 2.5. FreeSurfer Volumetric Segmentation

FreeSurfer analyzes and visualizes structural neuroimaging data [18]. The primary function of this system is to segment cortical and subcortical areas of the brain. Streams based on surface and volume undergo several steps in this process. A target image is transformed and corrected for intensity, and non-brain tissue is automatically removed. The target image is then mapped to an atlas using a high-dimensional nonlinear volumetric alignment. The cerebral cortex is segmented based on its gyral and sulcal structures to create a spherical atlas. Based on probabilities determined from a training set with manual labels, a subject-independent probabilistic atlas was constructed for each voxel to maximize its probability for the input signal.

The volumetric segmentation was performed using the FreeSurfer image analysis suite v6.0 (released in April 2017), which is documented and freely available for download online (http://surfer.nmr.mgh.harvard.edu/ in 31 December 2023.). All patient images were processed with the recon-all function (recon-all-i T1image-s sub-sd SUBJECTS/_DIR-all). The Desikan Killiany Atlas was used to calculate FreeSurfer volume estimates using the command asegstats2table for all subjects. This segmentation technique is based on a probabilistic atlas, and the Bayesian modeling approach is fully automated and can be found online [19]. Figure S2 shows that the corpus callosum (CC) was automatically divided into five subregions, ranging from the genu to the splenium, and categorized as anterior, mid-anterior, central, mid-posterior, and posterior sections, while the lateral orbitofrontal cortex (lOFC) was automatically divided into left and right lOFC. The “Volume” and “StructName” data from the segmentation were then extracted using the software R 4.3.3 for further analysis. A diagram of the experimental protocol was shown in Figure S3.

### 2.6. Statistical Analysis

All values are shown as means ± SD. Due to the relatively small group sizes, we used a non-parametric method to analyze data for the hippocampal subfield. Brain subfields from segmentation were assessed using the Mann–Whitney U test. We calculated Spearman’s correlation coefficients for the association between CC or lOFC volume change and MMSE score change to ascertain the relationship between brain subfield volume change and cognitive behavior. The unpaired Student’s *t*-test was used to compare the control and treatment groups. Statistical significance was defined as a *p*-value of ≤0.05.

## 3. Results

### 3.1. Safety Assessment After Transcranial Ultrasound Stimulation

Safety parameters were evaluated using brain MRI and contrast-enhanced MRI. Anatomical MRI assessments, including T2* and FLAIR imaging before and after TUS stimulation, showed no signs of intracerebral hemorrhage, structural brain abnormalities, microbleeds, superficial siderosis, BBB disruption, or other new intracranial pathologies. TUS treatment did not result in any serious clinical or radiographic adverse events in any of the patients.

### 3.2. Corpus Callosum Volume Change After Transcranial Ultrasound Stimulation

Table 2 shows that brain structures regarded as regions of interest increased in volume after TUS. The volume changes in the CC_Mid-Anterior, the CC_Central, the whole CC, the left lOFC, and the whole lOFC were significantly higher in the TUS group than in the placebo group (all *p* < 0.05). The other brain structures that significantly increased in volume after TUS are shown in Table S1.

**Figure 1:**
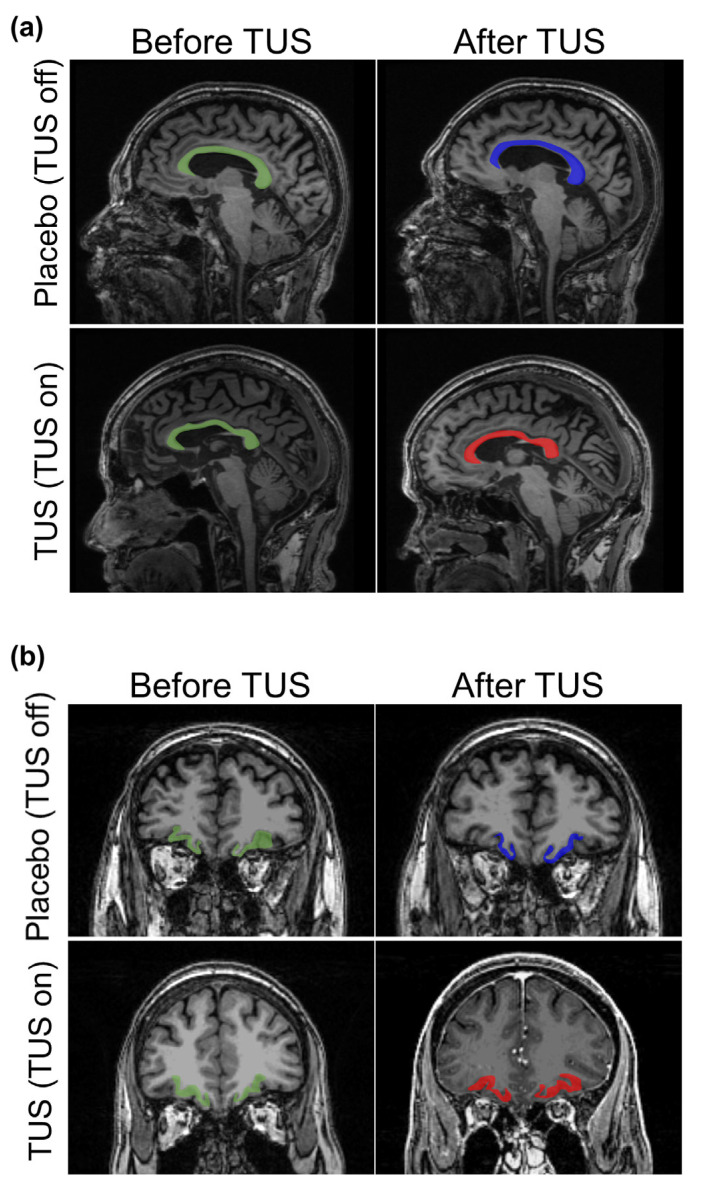
Representative T1-weighted magnetic resonance imaging (MRI) scan of patients with Alzheimer’s disease. (**a**) The corpus callosum before transcranial ultrasound stimulation (TUS) is highlighted in green. After TUS, the corpus callosum of the placebo group is highlighted in blue, while the corpus callosum of the TUS group is highlighted in red. First row represents the Placebo group. Second row represents the TUS group. For the detailed structure and function of the corpus callosum, please refer to Figure S2 in the supplementary file. (**b**) The lateral orbitofrontal cortex before TUS is highlighted in green. After TUS, the lateral orbitofrontal cortex of the placebo group after TUS is highlighted in blue, while the lateral orbitofrontal cortex of the TUS group is highlighted in red. First row represents Placebo group. Second row represents TUS group.

**Figure 2:**
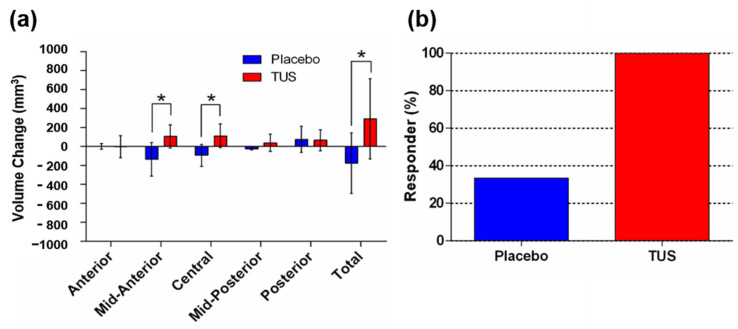
The effects of transcranial ultrasound stimulation (TUS) treatment on volume change in the corpus callosum. (**a**) Volume changes in each subfield of the corpus callosum. (**b**) Responder analysis of the effects of TUS; the proportion of corpus callosum responders was 100.0% (6/6) in the TUS group and 33.3% (1/3) in the placebo group. * denotes a significant difference at *p* < 0.05 between the placebo group (*n* = 3) and the TUS (*n* = 6) group.

The CC is a thick bundle of neurofibers connecting the two cerebral hemispheres. It has been found to undergo volume reduction in AD patients [20]. The change in CC morphology may lead to aberrant interhemispheric functional connectivity and memory and mild cognitive impairment [21,22]. The CC and lOFC regions recognized by FreeSurfer segmentation are shown in Figure 1a and Figure 1b, respectively. First, we examined the volume changes in the whole CC and the subregions. The results showed that the TUS group underwent more considerable volume changes than the placebo group in the whole CC, central CC, and mid-anterior CC (Figure 2a). To identify the TUS effect on CC volume change, we analyzed the responder rate of both groups. We defined the increase in CC volume as a positive response. The results showed a 100.0% positive response in the TUS group, while that of the placebo group was only 33.3% (Figure 2b). This suggests that TUS treatment potentially attenuates the decrease in CC volume in AD patients.

### 3.3. Lateral Orbitofrontal Cortex Volume Change After Transcranial Ultrasound Stimulation

**Figure 3:**
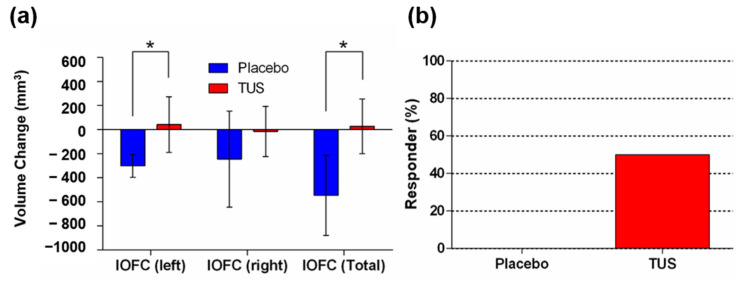
The effects of transcranial ultrasound stimulation (TUS) treatment on volume change in the lateral orbitofrontal cortex. (**a**) Volume changes in each lOFC subregion. (**b**) Responder analysis of the effects of TUS; the proportion of lateral orbitofrontal cortex responders was 50.0% (3/6) in the TUS group and 0.0% (0/3) in the placebo group. * denotes a significant difference at *p* < 0.05 between the TUS group (*n* = 6) and the placebo (*n* = 3) group.

In addition to the CC volume change, we observed a significant volume change difference in total lOFC and left lOFC between the TUS and placebo groups (Figure 3a). To identify the TUS effect on the lOFC volume change, we analyzed the responder rate of both groups. We defined an increase in lOFC volume as a positive response. The results showed a 50.0% positive response in the TUS group, while the placebo group showed a 0% positive response (Figure 3b). The lOFC is essential for controlling decision-making, negative emotions, and memory [23]. A previous study demonstrated OFC neurofibrillary tangle pathology in AD patients [24], and agitation behavior in these patients is thought to be related to this damage [25].

### 3.4. Relationship Between Volume Change and MMSE Score Change

**Figure 4:**
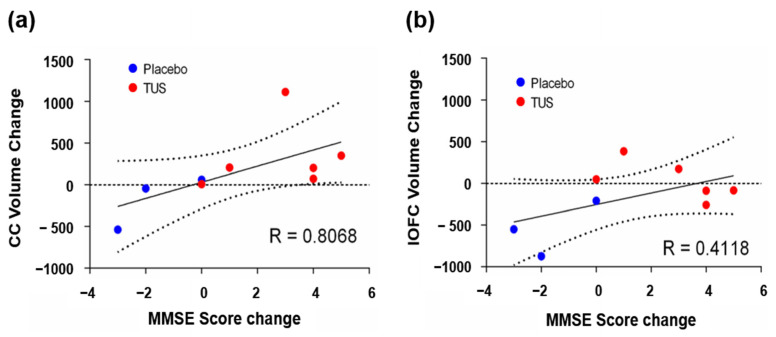
The correlation between ROIs volume change and MMSE score change after transcranial ultrasound stimulation (TUS). (**a**) Correlation between callosum volume change and MMSE score change. The Spearman correlation coefficient of 0.8068 indicates a strong positive correlation. (**b**) Correlation between lOFC volume change and MMSE score change. The Spearman correlation coefficient is 0.4118.

To investigate whether CC or lOFC volume change was correlated with MMSE score change, we used Spearman’s rank correlation. The changes in MMSE score of the subjects are shown in Table S2. In the case of the TUS group, the correlation between MMSE score change and CC volume change was significantly positive (R = 0.807, *p* = 0.012) (Figure 4a). These results suggested that CC volume change was highly correlated with MMSE score change. However, the MMSE score change did not show significantly correlation to the lOFC volume changes (R = 0.42, *p* = 0.271) (Figure 4b).

## 4. Discussion

We present LIPUS stimulation of the brain as a novel approach to the treatment of AD. Our results demonstrate that TUS treatment leads to significant changes in brain volume, particularly in regions such as the CC and the lOFC. In the TUS group, substantial volume increases were observed in the CC and the lOFC compared to the placebo group. Notably, the CC showed a 100% positive response in terms of volume increase, which was correlated strongly with improvements in the MMSE score, suggesting that LIPUS stimulation may enhance cognitive function by attenuating CC volume reduction in AD patients.

### 4.1. Non-Significant Change in the Volume of the Hippocampus

Previous research has extensively documented the essential role of the hippocampus in the progression of AD [26,27,28]. It is known to be one of the brain regions to be affected the earliest by AD pathology, which significantly contributes to the memory deficits and cognitive decline observed in patients [26,29,30]. TUS treatment for AD has also been reported to increase functional connectivity in the hippocampus [31]. Despite the recognized importance of the hippocampus, we did not find notable alterations in its volume of subfields in our study cohort (all *p* > 0.05). This observation is consistent with the suggestion of earlier studies that the involvement of the hippocampus may not always manifest as significant volumetric changes in all AD cases [32,33]. While hippocampal atrophy is a characteristic feature of AD, variability in its involvement has been noted across different individuals and stages of the disease [34]. The differences between our findings and those of earlier studies may be attributable to variations in the pathways and mechanisms involved in AD pathology, underscoring the need for a comprehensive examination of the various brain regions implicated in the disease process.

### 4.2. Some Brain Subfields Decreased in Volume After TUS

In contrast to the brain regions that showed significant volume increases after TUS treatment (Table S1), several brain subfields exhibited significant post-treatment volume decreases (Table S3). Specifically, we observed decreases in volume of the left anterior cingulate gyrus, left inferior opercular frontal gyrus, right inferior parietal lobule, right postcentral gyrus, and right insular white matter.

The anterior cingulate gyrus plays major role in social cognition behavior, that is, the ability of integrated social information from brain networks to support correct social interactions [35]. The cingulate gyrus was reported as having highly possible atrophy in AD patients. The anterior cingulate gyrus volume has even been shown to be twenty-percent smaller than control [36]. In AD patients, atrophy of the inferior opercular frontal gyrus can affect spatial perception and memory function; in addition, the inferior parietal lobule damage may affect language abilities and motor control, and its degeneration could lead to corresponding symptoms [37]. The postcentral gyrus is associated with tactile sensation and bodily awareness [38], and a reduction in the right postcentral gyrus was observed in AD patients [39]. Lastly, damages in insular white matter are found in AD patients, with neurofibrillary tangle and neuritic plaque throughout the insular cortex and white matter. This atrophy in AD patients may affect functional activity, leading to behavioral function impairments [40]. Although the reasons for these volume reductions remain uncertain, these changes may be attributable to AD, which is known to cause atrophy in various brain regions [41,42]. These findings highlight the complex nature of AD pathology and suggest that while some brain regions may respond positively to therapeutic interventions such as TUS, others may continue to exhibit atrophy as part of disease progression.

### 4.3. Potential Therapeutic Effects of TUS on AD Patients

TUS holds promise as a therapeutic intervention for AD patients. The observed effects on brain volume alterations, particularly in the CC and lOFC, suggest that it has potential for mitigating the progression of AD pathology. A previous study showed that TUS significantly increased functional connectivity in the hippocampus and improved Consortium to Establish a Registry on Alzheimer’s Disease (CERAD) neuropsychological scores [31], indicating potential attenuation of the brain volume reduction associated with AD pathology, thereby improving memory and cognitive function. Specifically, the CC, essential for interhemispheric communication and cognitive functions, exhibited a 100% positive response rate in terms of volume increase in AD patients who received TUS therapy. This increase in CC volume was strongly correlated with improvements in cognitive function, as assessed by the MMSE score. Moreover, changes in the volume of the lOFC, a region implicated in decision-making, emotional regulation, and memory, further suggest the therapeutic potential of TUS in preserving or enhancing cognitive functions affected by AD.

A limitation of this study is its small sample size of nine participants. This sample size may not represent the larger AD population and may limit the generalizability of the study’s findings. While the initial findings are promising, it is imperative to conduct further investigations involving larger study groups and longer observation periods to confirm the effectiveness of TUS in managing AD. It is essential to explore the mechanisms underlying the induction of brain volume changes and cognitive function improvements by TUS. This understanding is critical for optimizing the clinical application of TUS for treating AD.

## 5. Conclusions

After treatment with TUS, MRI scans of the brain subfields of AD patients showed enhancements. This observed effect was strongly associated with improvements in the MMSE score. This effect may stem from the mitigation of decreases in CC and lOFC volumes. The patients who underwent TUS showed improved CC and lOFC volumes, implying enhanced cognitive function.

## Figures and Tables

**Table 1:** Inclusion criteria.

Inclusion Criteria
Age between 55 and 90 years old.
Male or female.
Good understanding of written and verbal Chinese.
Geriatric Depression Scale (GDS) score of <8.
Mild AD patients: Mini-Mental State Examination (MMSE) score: 20–26.
“Probable AD” based on NIA/AA criteria.
The blood flow of bilateral middle cerebral artery (MCA) M1 can be detected by transcranial doppler (TCD) ultrasound.
Caregiver spending at least 10 h per week with the patient.
Agreement to obey the rules of this study.
Use of cholinesterase inhibitors for AD (e.g., donepezil, rivastigmine, etc.) will be allowed only if the participant has been on a stable dose for at least 6 months.
Use of antipsychotics will be allowed as long as the participant has been on a stable dose for at least 6 months.

**Table 2:** The volume change of Corpus Callosum and lateral orbitofrontal cortex in AD patients.

	Placebo			TUS						
StructName /Patients	1	2	3	1	2	3	4	5	6	*p*-value
CC_Anterior	−52.8	−161.6	22.2	198.8	−3.1	−14.6	32.6	−28.3	1.8	0.357
CC_Mid-Anterior	149	−58.8	−2.5	271.7	103.3	180.9	−339.6	−47.7	−21.9	0.048 *
CC_Central	93.5	21.3	10.6	336	36	178.3	−218.4	−66.3	7.4	0.012 *
CC_Mid-Posterior	25	192.3	−39.9	103.4	−35.5	−11.2	−11.9	−33.3	−25.7	0.274
CC_Posterior	−13.6	13.2	214.7	202.6	−29	15.3	−1.9	235.8	−7.2	0.452
CC_Total	−539.2	60.2	−45.6	8	6.4	205.1	1112.5	71.7	348.7	0.048 *
lOFC (left)	−211	−401	−287	42	−256	201	389	−124	5	0.024 *
lOFC (right)	−341	192	−587	−131	304	183	−217	−135	−89	0.191
lOFC (Total)	−552	−209	−874	−89	48	384	172	−259	−84	0.024 *

The table shows the volume alterations in CC and lOFC subfields for patients in the TUS group compared to the placebo group. Statistically significant changes (*, *p* < 0.05) are indicated for each region.

## Data Availability

The raw data supporting the conclusions of this article will be made available by the authors on request.

## Supplementary Materials

The following supporting information can be downloaded at: https://www.mdpi.com/article/10.3390/diagnostics15030359/s1, Table S1: The brain subfield volume increased of AD patients after TUS; Table S2: Changes in the Mini-Mental State Examination (MMSE) score of patients with Alzheimer’s disease after treatment with transcranial ultrasound stimulation (TUS). Table S3: The brain subfield volume decreased of AD patients after TUS. Figure S1: This figure shows that a Transcranial Ultrasound Stimulation (TUS) helmet for neuromodulation in patients, is used in brain research and medical applications. Figure S2: Subfields of the corpus callosum. Figure S3: A diagram of the experimental protocol for this study.: Changes in the Mini-Mental State Examination (MMSE) score of patients with Alzheimer’s disease after treatment with transcranial ultrasound stimulation (TUS).
