# Supplementary material for: Volume Changes in Brain Subfields of Patients with Alzheimer’s Disease After Transcranial Ultrasound Stimulation
Source: Diagnostics (Basel). 2025 Feb 4;15(3):359. doi: 10.3390/diagnostics15030359 (PMC11817765; doi:10.3390/diagnostics15030359)
Supplement: Supplementary file 1 [file diagnostics-15-00359-s001.zip › diagnostics-3404000-supplementary.pdf]

Table S1. The brain subfield volume increased of AD patients after TUS.

	Placebo			TUS						
StructName/Patients	1	2	3	1	2	3	4	5	6	*p*-value
Putamen (left)	127.6	-178.7	5.8	132.6	212	359.9	199.5	212.5	7.9	0.024*
Superior circular insula (left)	-111	-1675	-71	222	-57	-106	237	40	124	0.024*
Inferior precentral (left)	5	-78	-85	368	-11	56	374	173	91	0.024*
Anterior circular insula (right)	-53	-116	-45	-6	146	7	56	51	-52	0.024*
Intermedius primus Jensen (right)	-15	-105	40	174	117	27	62	49	107	0.024*
Visual cortex (right)	-244	-303	-158	-170	-5	103	-42	151	170	0.024*
Transverse temporal gyrus (right)	-16.2	-9.2	-9.3	11.7	6.6	40.2	43.5	4	-7.4	0.012*

The table shows the volume increase in various brain regions for patients in the TUS group compared to the Placebo group. Statistically significant changes (*, $p < 0.05$) are indicated for each region.

Table S2. Changes in the Mini-Mental State Examination (MMSE) score of patients with Alzheimer's disease after treatment with transcranial ultrasound stimulation (TUS).

Patient	MMSE Score Change
Placebo	
1	-3
2	0
3	-2
TUS	
1	4
2	0
3	1
4	3
5	4
6	5

Table S3. The brain subfield volume decreased of AD patients after TUS.

	Placebo			TUS						
StructName/Patients	1	2	3	1	2	3	4	5	6	*p*-value
Anterior cingulate gyrus (left)	411	214	81	11	-13	-350	-247	-44	71	0.024*
Inferior opercular frontal gyrus (left)	16	452	216	-116	-65	56	-43	-159	-179	0.048*
Inferior parietal lobule (right)	195	872	68	-447	-1517	-393	-1795	-296	15	0.024*
Postcentral gyrus (right)	92	-37	245	-77	-1008	-192	-97	-159	-67	0.024*
Insular white matter (right)	88.4	1345	384.2	-691.7	0	0.4	-127.6	-193.8	-580.2	0.036*

The table shows the volume decrease in various brain regions for patients in the TUS group compared to the placebo group. Statistically significant changes (*, $p < 0.05$) are indicated for each region.

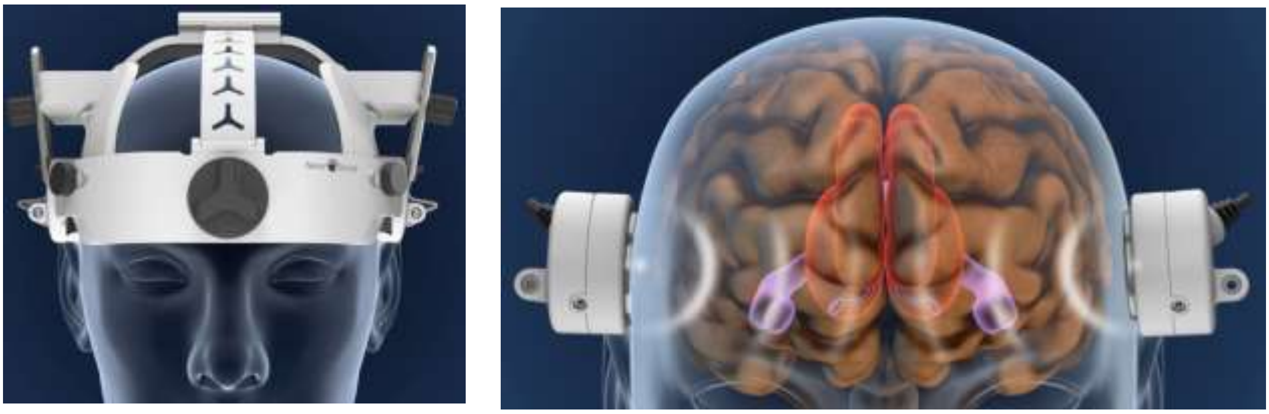

Fig. S1. This figure shows that a Transcranial Ultrasound Stimulation (TUS) helmet for neuromodulation in patients, is used in brain research and medical applications. Left Image: A head-mounted device with adjustable knobs and metal rods for precise positioning. Right Image: A transparent brain view highlights target areas like the amygdala, hippocampus, and frontal cortex in red and purple, indicating stimulation zones.

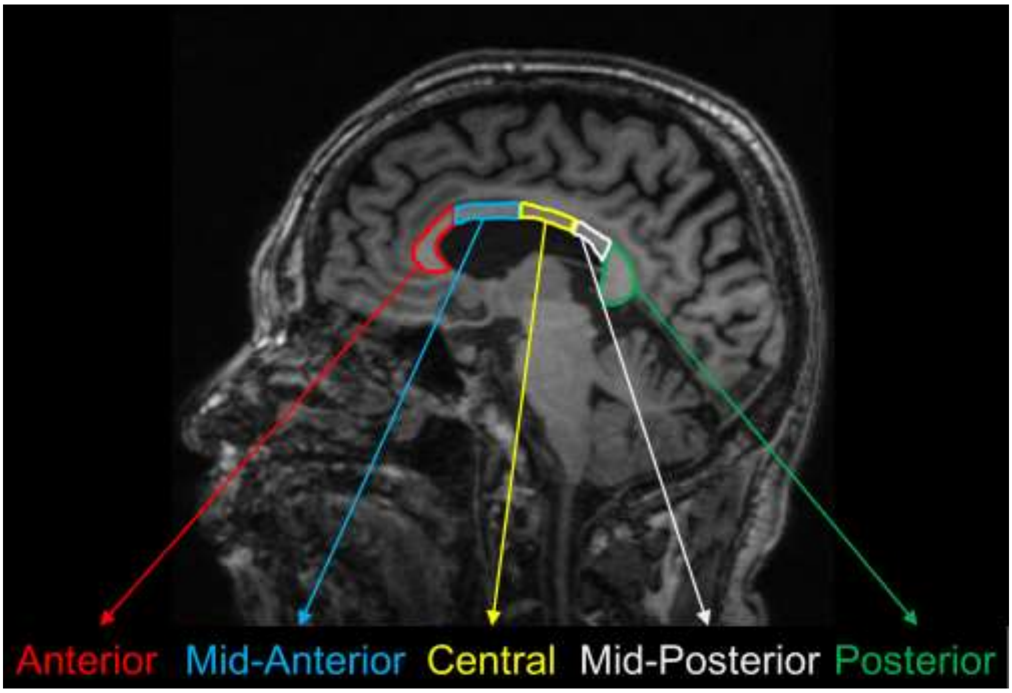

Fig. S2. Subfields of the corpus callosum. The corpus callosum was segmented into five subregions, ranging from the genu to the splenium, and categorized as anterior, mid-anterior, central, mid-posterior, and posterior sections. Functional and clinical relevance: The corpus callosum is the main connecting pathway between the cerebral hemispheres. Its degeneration is closely associated with cognitive decline in patients with Alzheimer's disease. Studying these detailed regions helps to understand how pathological changes affect specific cognitive or behavioral manifestations.

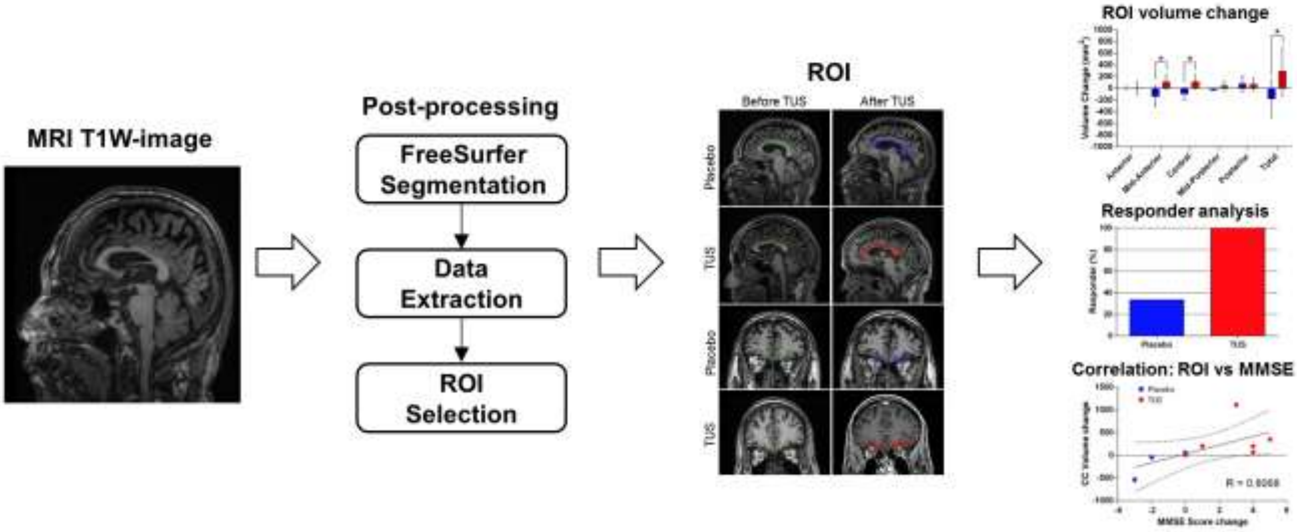

Fig. S3. A diagram of the experimental protocol for this study.

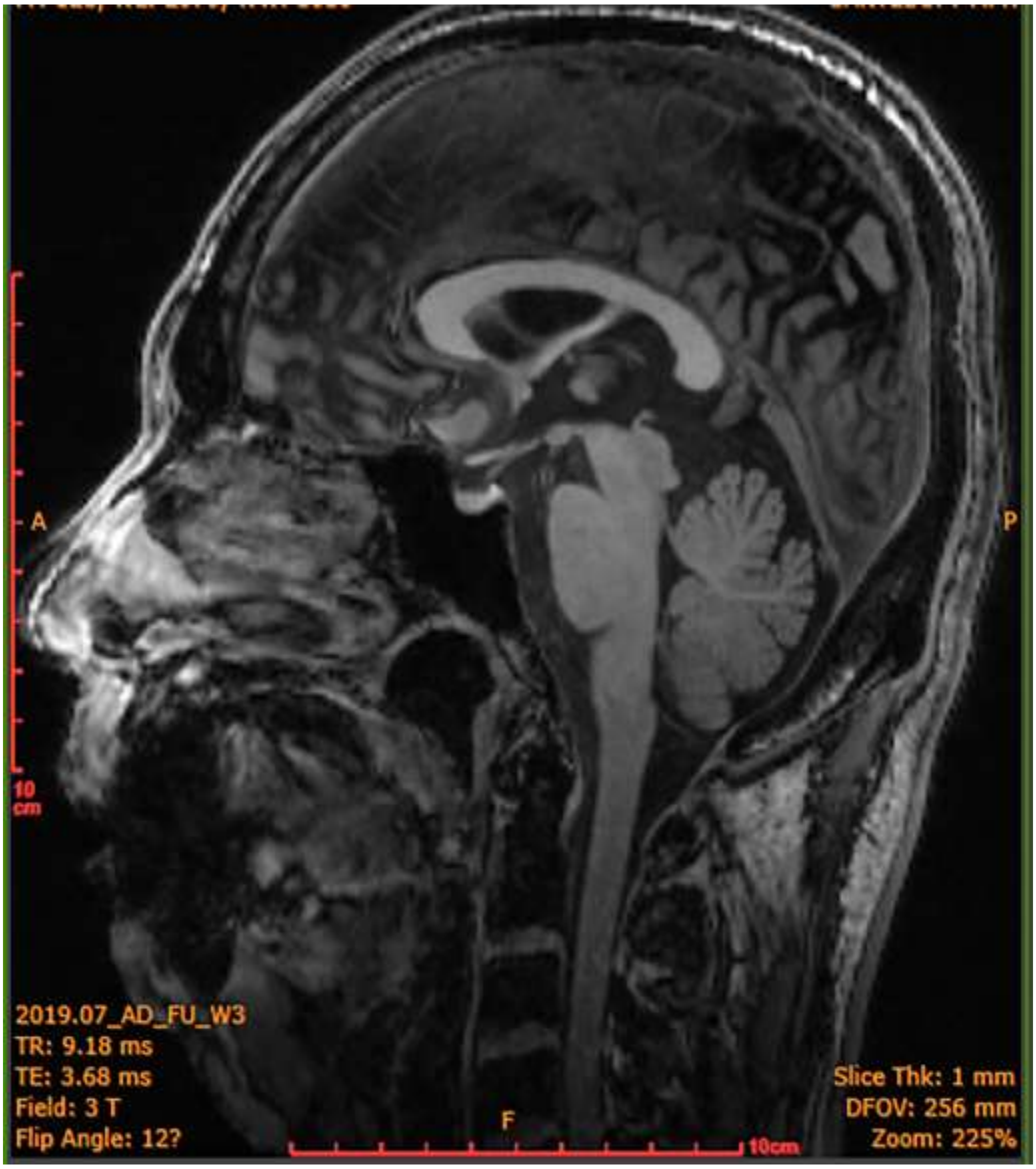

Fig. S4. MRI image.
